# Scientific Challenges to Free Will and Moral Responsibility

**DOI:** 10.1111/phc3.12200

**Published:** 2015-03-06

**Authors:** Joshua Shepherd

**Affiliations:** University of Oxford

## Abstract

Here, I review work from three lines of research in cognitive science often taken to threaten free will and moral responsibility. This work concerns conscious deciding, the experience of acting, and the role of largely unnoticed situational influences on behavior. Whether this work in fact threatens free will and moral responsibility depends on how we ought to interpret it, and depends as well on the nature of free and responsible behavior. I discuss different ways this work has been interpreted and argue that though work on conscious deciding and the experience of acting presents no real threat, work on situational influences is more difficult to dismiss. This work may present a real threat, and it may require us to revise our commonsense understanding of free and responsible behavior. But this work may also present ways to augment free and responsible behavior. Determining whether and how advancing science threatens, enhances, or simply describes free will is an ongoing task for scientists and philosophers alike.

## 1. Introduction

How is cognitive science relevant to free will and moral responsibility? In popular culture, and to some extent in both philosophy and cognitive science, two attitudes prevail. First, there is a general sense that at long last, the capacities that undergird free and morally responsible behavior are open to scientific enquiry. If we wish to understand free and morally responsible behavior – and perhaps if we wish to know whether we actually have free will – we are not stuck with philosophical reflection alone. Second, there is a general sense that current cognitive science threatens free will, and with it moral responsibility. What we are discovering about the mind is thought to undermine our free will, either by demonstrating that we do not actually have free will, or by demonstrating that we are in some way much less free than we thought.

What I say below primarily concerns this second attitude. Why think that cognitive science, or the result of any particular cognitive scientific experiment, threatens free will? In Section 2, I review a number of results that have been taken, in one way or another, to present a threat. In Section 3, I ask whether any of these results are as threatening as they are often purported to be. To preview, while talk of a threat from cognitive science is largely sound without fury, results highlighting the role of situational influences on behavior suggest that the folk psychological picture of agency is incomplete. Given this, it is possible that future science may uncover a genuine threat, and also possible that future science may uncover ways to augment free and responsible behavior. Clarity regarding either possibility requires clarity on the relationship between advancing knowledge in cognitive science and free and responsible action.

## 2. Purportedly Threatening Science

In this section, I review lines of research in cognitive science often taken, in one way or another, to threaten free will. Cognitive science is quite a busy enterprise, and as a result, the number of studies one might review under the heading ‘apparently threatening to free will’ is large. My review is selective. For ease of exposition, I will group the experiments under three headings. The experiments concern (a) conscious deciding; (b) the experience of acting; and (c) the role of largely unnoticed situational influences on behavior.

### 2.1. Conscious Deciding

Much of the worrying about a cognitive scientific threat to free will began in response to the work of neuroscientist Benjamin Libet and colleagues. The classic Libet study went as follows. Experimenters monitored electrical activity in the brains of participants, who sat watching a clock. A participant's task was to decide to flex a wrist, and to note the moment (on the clock) that an urge to flex appeared in experience.

Participants reported feeling an urge around 200 milliseconds before they began to flex. But about 550 milliseconds before the flex began, experimenters noticed an intriguing pattern of electrical activity in participants' brains. At this early stage, a negative shift in the so-called readiness potential (RP) – a pattern of activity typically associated with action preparation – could be detected. Why does this matter? Libet and colleagues interpreted things in this way: nonconscious neural activity had begun preparing the decision to flex at least 350 milliseconds before participants became aware of anything like preparation to decide.

Since onset of RP regularly begins at least several hundreds of milliseconds before the appearance of a reportable time for awareness of any subjective intention or wish to act, it would appear that some neuronal activity associated with the eventual performance of the act has started well before any (recallable) conscious initiation or intervention could be possible. Put another way, the brain evidently “decides” to initiate or, at the least, prepare to initiate the act at a time before there is any reportable subjective awareness that such a decision has taken place. (Libet et al. 1983, 640)

This result is perhaps startling enough as it is. But notice a prediction that follows from it. If nonconscious neural activity prepares the decisions we make well before we become consciously aware of these decisions, it should be possible to use early-stage neural activity to predict (or, on a certain interpretation of the neural activity, to decode) the decision. Recent work in the Libet tradition demonstrates that it is, in fact, possible to use such neural activity to predict (but not – at least not yet – to decode) decisions.

In a recent study by Chun Siong Soon and colleagues (Soon et al. 2013), participants watched a computer screen that displayed changing letters and numbers. Participants were instructed to spontaneously decide whether to add or subtract the passing numbers, and to memorize the letter present when they made this decision. This allowed the experimenters to locate the time of the decision.

Up to four seconds before the decision, Soon et al. found neural activity in the medial frontopolar region of the brain that predicted the upcoming decision (to add or subtract) with 59.5% accuracy, as well as neural activity in the precuneus and posterior cingulate that predicted the upcoming decision with 59% accuracy. It is important to note that these percentages are highly constrained by the relative crudity of the brain-monitoring technology we currently have, as well as the relative crudity of our understanding of the nature of decision-making activity in the brain. There is at least some reason to expect that improvements in technology and understanding will push these percentages higher.

### 2.2. The Experience of Acting

There is much work on the experience of acting that no one takes to threaten free will. The work that is taken to threaten free will is so taken in large part because it suggests that the experience of acting is at times illusory. In one much-discussed study, Daniel Wegner and Thalia Wheatley (1999) had participants engage in a joint action with an agent who was surreptitiously in collaboration with Wegner and Wheatley. Both participant and confederate faced each other at a table. Both placed their hands on a wooden square that sat atop a computer mouse. The movement of the wooden square moved a cursor on a screen populated with various objects. Both participant and confederate wore headphones that, in the participant's case, played words and music. The words were purportedly random distractors; the music indicated when participants were to stop the cursor. The truth: Wegner and Wheatley used the words – which named objects on the screen – to prime participants regarding the stopping of the cursor.

What was the point of the confederate? In one condition, the confederate exerted influence over the stopping of the cursor. In a second condition, the confederate allowed the participant to stop the cursor unfettered. After bringing the cursor to a stop, participants gave a rating on a 0–100 scale, with 0 indicating ‘I allowed the stop to happen’ and 100 indicating ‘I intended to make the stop.’

In the unfettered condition, participants gave an average rating of 56. But in the condition in which the confederate exerted influence over where the cursor stopped, the average rating differed depending on when the participant heard a word that matched the eventual stopping point of the cursor. When participants heard a matching word 30 seconds before the stop, they gave an average rating of 44. But when participants heard a matching word 5 seconds before the stop – a time close enough to plausibly prime the participant with an expectation of stopping the cursor there – they gave an average rating of 60. This difference of 16 points is statistically significant. Wegner and Wheatley concluded that ‘the experience of will can be created by manipulation of thought and action… and this experience can occur even when the person's thought cannot have created the action’ (1999: 489).

In a similar study, Wegner, Sparrow, and Winerman (2004) had participants sit facing a mirror. Participants wore headphones, along with a smock and gloves. Participants kept arms at sides; a confederate placed arms with gloved hands through the arm-holes of the smock. As a result, the hands the participants saw in the mirror looked very much like their own. Over a period of minutes, the confederate performed a number of actions with the hand while the participant watched. One group of participants heard nothing through the headphones. A second group heard a series of instructions (e.g., ‘hold up your left hand and spread the fingers apart,’ ‘wave hello with your right hand’) as the confederate performed the relevant actions.

After this, participants gave a rating to several questions. Two relevant questions were these: How much control did you feel that you had over the arms' movements? To what degree did you feel you were consciously willing the arms to move? In response to these questions, and again on average, participants who heard the instructions as the actions were performed, gave a rating of 3.00 on a 1–7 scale (with 7 representing the maximum amount of control or conscious will). Participants who heard nothing gave a rating of 2.05 on a 1–7 scale. The difference between these ratings is statistically significant. Wegner, Sparrow, and Winerman comment: ‘The curious implication of the research is that the experience of control over one's own movements is potentially open to extension to an experience of control over anything at all. Perhaps all that is needed for the development of a sense of agency is a preview that allows the person to establish a sense of mental causation over the previewed event.’ (845)

### 2.3. The role of Largely Unnoticed Situational Influences on Behavior

A massive literature in social psychology – roughly grouped under the title ‘situationist social psychology’ – offers evidence that non-obvious features of situations influence human behavior in a wide and surprising range of ways. There is no way to do justice to this literature in a short paper.^1^ Here, I will very briefly present the results of five interesting experiments. There are many more in the neighborhood.

First, participants sat in a room alone. They believed they were talking to another person or persons about the difficulties of being a college student, when in fact they were hearing a recording of a confederate. The confederate said they felt they were about to have a seizure, began to speak incoherently, said that they were afraid they might die, and eventually began to make choking sounds, at which point the recording would end. Participants had 125 seconds to intervene before the recording ended. The main result of this experiment is the following: 85% of people who believed they were alone got up to intervene before the recording ended, 62% of people who believed another person was nearby got up to intervene before the recording ended, and 31% of people who believed five others were nearby got up to intervene before the recording ended (Darley and Latane 1968).

Second, participants were presented with noise levels of either 85 dB or 65 dB. Participants presented with louder noises were less likely to help a nearby person who dropped a belonging (Matthews and Cannon 1975).

Third, people presented with pleasant smells (e.g., those of a bakery or coffee shop) are more likely to help in response to a request to help than are people presented with no pleasant smells at all (Baron 1997).

Fourth, people wearing sunglasses behave more selfishly than those not wearing sunglasses (Zhong et al. 2010).

Fifth, participants in a psychology department voluntarily deposited almost three times as much money in the departmental ‘honesty box’ (used to pay for coffee, tea and milk) when an instructional sheet above the box included an image of a pair of eyes (as opposed to an image of flowers) (Bateson et al. 2006).

As should already be apparent, the unnoticed situational factors appear to influence behavior in a startlingly wide range of ways: ways that prompt many social psychologists to think of the production of human behavior in terms of ‘the power of the situation’ rather than the power of the agent. Matthew Lieberman summarizes: ‘If the power of the situation is the first principle of social psychology, a second principle is that people are largely unaware of the influence of situations on behavior, whether it is their own or someone else's behavior’ (2005, 746).

### 2.4. The Nature of the Threat

With respect to human agency, these results tell a similar story. Roughly, the story is this. Our conscious awareness of agency does not tell the full story about agency, and in many cases, our conscious awareness of agency might tell a lie. Work on conscious deciding has been taken to show that our experience of consciously deciding is illusory – what really happens is that nonconscious processes settle matters before we are ever aware that matters are settled. Work on the experience of acting has been taken to show that conscious experience is causally unimportant to the production of many actions. It might seem as though we consciously initiate, guide, and sustain our actions, but in fact we simply experience the initiation, guidance, and sustenance after the fact. Work on the role of unnoticed situational factors has been taken to show that the commonsense understanding of action, on which we assess and decide to act on the basis of consciously recognized reasons, is misleading. In fact, many of the features that move us to act do so via non-conscious, reasons-irrelevant processes.

These are all claims based on interpretations of a series of results. Most would agree that if correct, these claims, and the evidence taken to license them, present a threat to free will and moral responsibility.^2^ What we want to know, of course, is whether the evidence licenses the claims.

## 3. Is There an Actual Threat Here?

Let us take the results in order.

### 3.1. Conscious Deciding

Regarding conscious deciding, Libet and others have taken the relevant data to license the view that either nonconscious neural processes determine our decisions well before the agent becomes aware of the content of the decision, or nonconscious neural processes begin to prepare an agent's decision well before the agent becomes aware of the preparations. Is either interpretation correct?

For all we currently know, the answer is no. First, as Alfred Mele (2009) has convincingly argued, it is doubtful that the above studies on their own support the strong interpretation (see also Bayne 2011 for an excellent discussion). For the above studies at best offer evidence that neural processes that are not identical with an urge to decide (the urge reported roughly 200 milliseconds before wrist movement) are involved in the preparation of a decision. It remains possible, however, that whatever decision agents make is made consciously. Indeed, Mele (2009) reviews work on reactions times that indicates Libet's data in fact leave enough time for participants in these studies to acquire intentions to flex and for these intentions to initiate the flexing within the 200 milliseconds after an urge to flex arises.

Libet's interpretation of the data depends on an important assumption: that the negative shift in the readiness potential that begins about 550 milliseconds before a decision is made reflects preparation to decide. But recent work gives us a reason to question this assumption. As background to this work, notice that the kinds of decisions made in Libet's experiment are more-or-less meaningless to the participants. Nothing hangs on when a participant decides to flex; there is no reason to decide to flex at time t1 rather than at time t2 (on this and related points, see Mele 2009, Roskies 2010, Waller 2012). So it is possible that the kind of neural processes that underlie decisions to flex are different in kind from the processes that underlie decisions on the basis of reasons.

Aaron Schurger and colleagues (2012) argue that the readiness potential is better understood not as preparation to decide but rather as a reflection of neural noise, the kind of gradual increase in neural activity that precedes many spontaneous movements, but is not present in all cases of intentional movements. Schurger et al. summarize as follows:

We suggest reserving the term “decision” to the commitment to move achieved once neural activity (spontaneous or goal directed) crosses a specific threshold… The reason we do not experience the urge to move as having happened earlier than about 200 ms before movement onset is simply because, at that time, the neural decision to move (crossing the decision threshold) had not yet been made. A very similar fluctuation in neuronal firing could equally well, at some other time, have not preceded the movement… We propose that the neural decision to move coincides in time with average subjective estimates of the time of awareness of intention to move and that the brain produces a reasonably accurate estimate of the time of its movement-causing decision events. (E2910)

Of course, this point does not generalize to studies like Chung Siong Soon's. Soon and colleagues do not rely on readiness potentials to predict an agent's decision. Even so, as Mele (2012) and others have noted, the kind of neural activity Soon and colleagues identify might simply reflect early biases or preferences in the decision-making process. It is not terribly surprising to discover that at time t1, I have a 60% (or even an 80%) bias towards deciding to add rather than deciding to subtract two random numbers. After all, my decision might be influenced by what I did in the past, by which operation seems more interesting, or by any number of things. Regarding studies on conscious deciding, what we want to know is whether (and to what extent) decision-making processes in the brain are available to consciousness, and whether (and to what extent) availability to consciousness is functionally important for the process.^3^

Although we lack detailed answers to these questions, nothing in the Libet tradition suggests that consciousness is irrelevant to decision-making. Indeed, some evidence suggests that at least regarding some decision-making processes, conscious experience provides a fairly accurate indication of the nature of the process. Return to a Libet-style flexing case. Han-Gue Jo and colleagues (Jo et al. 2014) had an expert meditator go through a Libet-style experiment. In one condition, they asked the participant to notice when an urge to flex appeared in consciousness, and then to wait for as long as possible before flexing. When this was done, the RP showed a similar pattern before the urge appeared, but crucially, the RP did not immediately cause the wrist flexing. Instead, the participant waited for up to 3 seconds before flexing. It is plausible, then, that the RP reflects neural preparation not for a decision, but for the appearance of an urge to move in consciousness – an urge that at least some participants are free to follow or reject.

### 3.2. The Experience of Acting

I turn to data on the experience of acting. Some – most famously Daniel Wegner (2002) – have argued that this data support a model on which the experience of acting is systematically illusory. But this interpretation of the data has been soundly criticized in a number of places, such that at this point, there is very little to recommend it.^4^ Taken most charitably, the studies discussed in Section 4 demonstrate that aspects of our experience of acting – for example, whatever aspect is picked out by responses to the question ‘To what degree did you feel you were consciously willing the arms to move?’ where the agent knows the arms were not hers – are malleable. It is doubtful that this on its own threatens free will. Consider, by analogy, that visual experiences are malleable in a wide range of ways. But no one should believe that visual experiences are systematically illusory. In fact, visual experiences are generally quite accurate.

Subsequent work on experiences of acting indicates something similar for experiences of acting. The best current models for such experiences hypothesize that the mechanisms responsible for experiences of acting take as input multiple cues and utilize a multifactorial process of weighing relevant cues to produce an optimally accurate experience (Synofzik et al. 2008; Moore and Fletcher 2012). Relevant cues might include an agent's intentions, an agent's expectations, lower-level motor commands, predictive states, error signals, and also various perceptual states related to the progress of the action. Importantly, this kind of multifactorial weighting of cues means that experiences of acting will often contain little information about the fine-grained motor adjustments agents often make while acting. Instead, it seems that agents experience the progress of their own actions at a relatively rough-grained level.

Why would experiences of acting be geared towards accuracy at such a level? Here is one plausible proposal. We know that the mechanisms at work in action control are organized hierarchically, with lower-level mechanisms subserving the implementation of fine-grained, highly specific motor programming and adjustment, and with higher-level mechanisms subserving the implementation of rough-grained, more abstract elements of action plans (see Grafton and Hamilton 2007, Logan and Crump 2011, Shepherd forthcoming). The higher-level mechanisms need an accurate picture of what is happening at a certain level of abstraction if they are to flag errors and make adjustments at that level of abstraction. It appears that experiences of action serve just this function, enabling an agent to notice large-scale errors and make adjustments to action-plans that fit her on-going perception of the environment. If so, the data Wegner and colleagues offer is not threatening to free will, even though it indicates that experiences of action are not infallible.

### 3.3. The Role of Largely Unnoticed Situational Influences on Behavior

Regarding the relevance of situationist evidence to free will and moral responsibility, at least two questions arise. First, how is it that these results present a threat? Second, how serious is the threat? If it turns out that the threat is serious, of course, we may face practical questions regarding how we ought to adjust our practices of holding others responsible for their conduct. But in what follows, I focus primarily on the first two questions.

In an influential paper, Dana Nelkin considers the first question at some length. While there are several ways that individual situationist experiments might seem threatening, ultimately Nelkin finds a common theme uniting the situationist evidence: ‘simply put, the subjects [in the experiments] seem to be acting for bad reasons, or at least not acting for good reasons, and they seem stuck doing so. At the same time, having the ability to act for good reasons is essential to freedom and/or responsibility’ (2005, 199). Subsequent discussions have largely agreed with Nelkin on this point. The situationist evidence is considered threatening to the extent that it undermines our conception of ourselves as agents who recognize the reasons for action that are present in a situation, and as agents who have the ability to act on the basis of the recognized reasons.

This answer to the first question suggests a way to think about the second question. In short, the threat from situationism will be serious to the extent that it undermines the view that agents adequately recognize and respond to reasons for action. Given the fact that we lack the full empirical story regarding reasons-sensitivity, it is perhaps unsurprising that philosophers disagree about the relevant extent.

Consider David Brink's view. Brink (2013) reckons that the relevant evidence shows that on occasion, the capacities that undergird morally responsible agency are masked or thwarted in a way that undermines moral responsibility. But for Brink, this only happens at the margins (i.e., in cases of wartime conduct^5^): for Brink, ‘in paradigmatic contexts studied by situationists there is little reason to deny that agents are responsible for their conduct’ (123).

Why think this? Brink notes that moral responsibility for an action is predicated on the possession, rather than the use, of capacities to recognize and respond to reasons in favor of or against that action. An agent who is competent in a situation is responsible for her performance, even if we have reason to believe that situational factors negatively influenced her performance. And Brink argues that the role of situational factors on behavior production is most plausibly interpreted as impacting performance (that is, use of the relevant capacities), rather than competence (that is, possession of the relevant capacities).

Brink offers a few considerations in favor of the claim that we retain capacities for appropriate action even when situational factors undermine the appropriate use of these capacities. First, Brink notes that although many participants in the situationist experiments seem to misbehave, many in fact do the right thing. This suggests that capacities for appropriate action are robust against situational factors for at least some agents. Second, in at least some of the situationist experiments, many of those who misbehave show signs that doing so is uncomfortable or stressful (e.g., Milgram 1963). This suggests, to Brink, that the misbehaving agents retain the relevant capacities. Third, Brink speculates that seemingly very similar circumstances might engage these capacities. Concerning a famous study in which participants administered seemingly painful shocks to other people at the request of an experimenter, Brink asks: ‘Would the compliant subjects have administered serious shocks if they had been given more time to consider their options, if they had been asked to justify the imposition of apparent harm to innocent parties…?’ (141) Brink thinks the answer is yes, and that this answer indicates the retention of the relevant capacities.

However, whether agents retain capacities for appropriate action depends in part on the right way to individuate and identify the relevant capacities. Regarding identification, signs of discomfort and stress are not clearly evidence for a capacity for appropriate action. So it is not clear that this consideration cuts much ice.^6^ Regarding individuation, the fact that some people act rightly might simply indicate that in some circumstances, some individuals retain capacities that others do not. Compare the difference between myself and four-time Olympic gold medallist alpine ski racer Janica Kostelic. We both have the capacity to ski very steep hills, but this is not very informative. Kostelic can ski much steeper hills at much greater speeds with much more grace and fluidity. Arguably, even if they exist together on a continuum, Kostelic's capacities for excellent skiing are much different than mine, such that she retains a capacity to ski steep hills at high speeds in situations – high-pressure competition, icy hills, etc. – that I do not. The same might be true of capacities for right action: there may be a morally important difference between the capacities of those who act rightly and those who do not in the situationist experiments.

A similar point holds regarding Brink's claim that changing circumstances might plausibly lead to right action. It is possible that doing so marks a morally important difference, in virtue of the fact that changing circumstances engages capacities for right action in a morally salient way. One thing the situationist evidence might show is that folk psychological distinctions regarding our capacities are insufficient to fully explain behavior. It may be that we need to individuate capacities at a finer level in response to the situationist evidence.

Something like this view is urged by Manuel Vargas (2013). Further, Vargas argues that a view of free and responsible agency consistent with the role of the situation is available (whether we should adopt it is a question beyond the present scope). On this view,

The range of moral considerations an agent recognizes in some or another context or circumstance will vary. In some circumstances agents will be capable of recognizing a wide range of moral considerations. In other circumstances those sensitivities may be narrower or even absent. When they are absent, or when they dip beneath a minimal threshold, the agent ceases to be a responsible agent, in that context. (2013, 337)

Some will find Vargas's situation-specific view of morally responsible agency quite attractive.^7^ But it is important to note that on this kind of account, we have conceded a fair amount to situationist critics of agency. This kind of account is a departure from commonsense regarding morally responsible agency, and as such, it is sure to meet criticism. Whether a situation-specific view is the right one requires further examination of emerging empirical work, as well as further examination of the philosophical arguments and assumptions that undergird such a view.

## 4. Conclusion

We want to know whether cognitive science threatens free will and moral responsibility (and if so, how and why). Unsurprisingly, the answer requires nuance. Research paradigms focusing on conscious deciding, and on the experience of acting, do not (at present) present a genuine threat. But research on the role of largely unnoticed situational influences on behavior does present a kind of threat. This research does not seem to undermine the very existence of free will, but it does seem to undermine what we might call a Reasons-Driven View.

Reasons-Driven. In any given circumstance, the capacities that undergird free and responsible behavior track almost all relevant reasons for action, almost always successfully implement rational plans of action, and do so untainted by low-level, reasons-irrelevant mechanisms and processes.

One might complain that this undermined view is pretty naïve. We readily admit that stress, exhaustion, and various negative emotions impair our reasons-relevant capacities. Even so, the situationist evidence remains surprising. For this evidence suggests that *at least some* actual human behavior does not even approximate this admittedly naïve view. Yet most of us – even the sophisticates among us – hope that we approximate this view. Otherwise, it is difficult (even if possible) to make sense of our practices of holding one another responsible for our behavior.

The notion of approximating this view is of course a degreed one. And this raises a number of difficult issues. Is the degree of reasons-sensitivity we actually possess sufficient for some level of moral responsibility? Where is the level of reasons-sensitivity beyond which freedom and responsibility is mitigated? Where is the level beyond which it no longer exists? Facing such questions, it seems paramount to determine with some accuracy our actual levels of reasons-sensitivity, as well as the kinds of circumstances that tend to undermine or enhance these levels. For in spite of a wealth of data on the role of situational influences, theorizing regarding the behavioral mechanisms via which situations do their subterranean work – as well as how these mechanisms interact with reasons-sensitive mechanisms – is in its infancy. An actual understanding of the extent of the threat from situationism, then, looks to require a good deal more cognitive science, a good deal more philosophy of mind, and with these, a good deal more moral philosophy.

In this connection, it is worth mentioning an optimistic reading of the situationist evidence. Notice that even if situations impact behavior in unnoticed ways, there is no reason to think that their impact is entirely negative. Certainly, immoral behavior gets more press, but we should expect that if situational influences sometimes impair moral or rational behavior, situational influences sometimes enhance moral or rational behavior (see Sarkissian 2010, Vargas 2013). Determining when and why behavior is enhanced would seem, then, to progress alongside an understanding of when and why behavior is impaired. Perhaps there is some in principle reason that situations tend to impair behavior more than they tend to enhance it. But given what we currently know, I cannot see the reason. It is possible that the threat of situationism, while real, gets far more attention than it should. Perhaps the situationist evidence is full of both peril and promise regarding free and responsible behavior.

## Short Biography

Joshua Shepherd is a Wellcome Trust Research Fellow at the University of Oxford, a Junior Research Fellow of Jesus College, and a James Martin Fellow at the Oxford Martin School. He received his PhD from Florida State University. He works on issues in the philosophy of mind, action, cognitive science, and moral psychology, and is especially interested in agency, in consciousness, in relationships between agency and consciousness, and in the normative significance of consciousness.

## Footnotes

1 There has been a robust debate over whether the situationist literature demonstrates that human agents lack character traits, but as this is tangential to concerns about free will and moral responsibility, I do not consider that debate here (see Doris 2002, Srinivasan 2002, Alfano 2013, Murray 2014). For discussions of this literature, and its relevance to free will and moral responsibility, see Brink 2013, Ciurria 2013, Nelkin 2005, Vargas 2013, Mele and Shepherd 2013.

2 For work in experimental philosophy demonstrating the importance of consciousness, including conscious deciding, to folk views on free will and moral responsibility, see Shepherd (2012). Here, I am putting aside one line of response available to the free will and moral responsibility defender – a line that accepts all or some of the above claims and argues that even so, free will and moral responsibility are intact. It is interesting to reflect on how this line of defense might best be articulated (for one way to articulate this line of response, see Mele (2013)), but I say nothing more about that here.

3 Some have challenged the widely held view that deciding is an intentional action (see Strawson 2003). If not, of course, views of free action that focus heavily on deciding might be in trouble. For a recent defense of the view that deciding is an intentional action, see Shepherd (2014a).

4 For criticisms, see Nahmias 2002, Bayne 2006, Shepherd 2013, Walter 2014. For arguments that Wegner's work (among others) favors skepticism about free will, see Caruso 2012. For positive proposals about the role of consciousness in action, see Shepherd (forthcoming-b), Tse (2013).

5 For more on the relevance of the situationist literature to the issue of moral responsibility for wartime conduct, see Doris and Murphy (2007).

6 An anonymous referee notes that in some cases signs of discomfort and stress may well indicate that the agent recognizes reasons against the action they are performing. (They may not, however. The relationship between signs of discomfort and the recognition of reasons not to do what one is doing strikes me as delicate and worth further consideration.) This same referee notes, rightly in my view, that while recognizing reasons not to A is relevant to a capacity to not A, these are two separate things.

7 For a congenial view of the nature of control over action that emphasizes the importance of circumstances, see Shepherd (2014b).
